# Design of Thermoplastic 3D-Printed Scaffolds for Bone Tissue Engineering: Influence of Parameters of “Hidden” Importance in the Physical Properties of Scaffolds

**DOI:** 10.3390/polym12071546

**Published:** 2020-07-13

**Authors:** Nieves Cubo-Mateo, Luis M. Rodríguez-Lorenzo

**Affiliations:** 1 Sensors and Ultrasonic Systems Department, Institute for Physical and Information Technologies, ITEFI-CSIC, 28006 Madrid, Spain; nieves.cubo@csic.es; 2 Department of Polymeric Nanomaterials and Biomaterials, ICTP-CSIC, 28006 Madrid, Spain

**Keywords:** polycaprolactone, 3D printing, scaffolds, bone engineering, thermo-mechanical properties

## Abstract

Additive manufacturing (AM) techniques are becoming the approaches of choice for the construction of scaffolds in tissue engineering. However, the development of 3D printing in this field brings unique challenges, which must be accounted for in the design of experiments. The common printing process parameters must be considered as important factors in the design and quality of final 3D-printed products. In this work, we study the influence of some parameters in the design and fabrication of PCL scaffolds, such as the number and orientation of layers, but also others of “hidden” importance, such as the cooling down rate while printing, or the position of the starting point in each layer. These factors can have an important impact oin the final porosity and mechanical performance of the scaffolds. A pure polycaprolactone filament was used. Three different configurations were selected for the design of the internal structure of the scaffolds: a solid one with alternate layers (solid) (0°, 90°), a porous one with 30% infill and alternate layers (ALT) (0°, 90°) and a non-alternated configuration consisting in printing three piled layers before changing the orientation (n-ALT) (0°, 0°, 0°, 90°, 90°, 90°). The nozzle temperature was set to 172 °C for printing and the build plate to 40 °C. Strand diameters of 361 ± 26 µm for room temperature cooling down and of 290 ± 30 µm for forced cooling down, were obtained. A compression elastic modulus of 2.12 ± 0.31 MPa for n-ALT and 8.58 ± 0.14 MPa for ALT scaffolds were obtained. The cooling down rate has been observed as an important parameter for the final characteristics of the scaffold.

## 1. Introduction

Additive manufacturing (AM) techniques are becoming the techniques of choice for the development of scaffolds in tissue engineering (TE). The main advantage of AM over formerly used techniques, such as freeze-drying, particulate-leaching, electrospinning, thermally-induced phase separation or cryopolymerization [1,2,3,4], is the ability of designing geometrical parameters of the scaffolds such as pore size, pore strut thickness, pore interconnectivity and pore morphology in advance, and the potential to tailor and scale the manufacturing of scaffolds to each experimental model or patient. In addition, 3D printing has significantly increased the economic feasibility of low volume production runs, because the majority of investment for traditional manufacturing methods like injection moulding is for set up (e.g., fixturing, tooling, and moulds) and costs can only be recouped for high volume production runs [5]. However, the development of 3D printing for tissue engineering brings unique challenges such as process variability that must be accounted for in the design of experiments.

Tissue engineering scaffolds should have similar mechanical properties to native tissue for withstanding similar physiological loading, which is a critical parameter for the regeneration of the target tissue. For bone, mechanical properties should be achieved in combination with high porosities that allow cell migration [6], vasculature growing [7] and a degradation kinetics of the material that eventually facilitates the substitution of the implanted device for new ingrowth tissue [8]. Thus, manufacturing processes of these scaffolds should be precise to achieve such a demanding working condition.

The printing process parameters must be considered as effective and important factors in the quality of final 3D-printed products [9]. A correlation between architecture and compressive modulus, regardless of the formulation tested, has been observed, which demonstrates how the laydown pattern influences the resulting 3D-printed scaffolds’ stiffness [10]. Depending on the technique selected, these parameters include pressure and printing speed, geometrical specifications, the path length (each straight line in the printing pattern), the path direction (the angle of the path with the vertical axis of the printing bed), and the printing direction (right to left and front to back). All these specifications of a scaffold have a direct effect on its biological and mechanical properties and some of them have been studied with the help of finite element analysis (FEM) [11]. Other parameters mentioned in several publications but not frequently described are: the influence of layers on the mechanical properties, in particular the induced anisotropy where the boundary between adjacent layers represents weak regions with maximum residual stresses [12]. The spatial resolution, which has a dramatic influence on the functionality of printed objects. The printing fidelity from the CAD design, related many times with the printing conditions, rheological behaviour and printing and bed temperature. The spatial resolution depends also on a precise positioning of the machine during the scaffold fabrication process [12]. In summary, the parameters determining scaffold properties are complex and frequently not sufficiently described in the literature related to tissue engineering, where the focus is usually on the biological response [13,14,15].

Scaffold design also requires the control of material properties, surface topography, chemistry, and stiffness that significantly influences biological processes [16]. Polycaprolactone (PCL) is one of the most used synthetic polymers for tissue engineering and regenerative medicine applications. This is due to the appropriate properties of PCL. These include long-term biodegradability, ease of processing due to the low melting temperature (≈58–60 °C), suitable rheological and viscoelastic properties, and thermal stability. Because of its history in drug delivery devices, PCL has also a shorter regulatory path to market than many other polymer systems through the Food and Drug Administration (FDA) and the European Medicines Agency (EMA). As an example, a custom designed airway splint device was printed using PCL, and administered to the patient under the emergency-use exemption from the FDA [17]. Although PCL is not bioactive, the introduction of active biological clues does not require strong synthetic efforts. It can easily be manufactured with properties tailored to suit specific applications [18,19]. Some groups have also already worked with PCL in combination with living cells to create hybrid constructs for bone or cartilage tissue engineering [20,21,22].

For the field of TE, AM techniques can be grouped into acellular techniques where cells cannot be incorporated into the manufacturing process, and bioprinting where living cells can be included in the precise placement of material during fabrication [23]. Bioprinting involves the use of bioinks, which combine biological and hosting synthetic materials [24]. Hydrogels are normally used as the hosting material for bioinks but they do not present appropriate mechanical properties for bone or cartilage applications [25]. Bone tissue engineering efforts have been, then, directed to combine both approaches, acellular scaffolds and bioprinting. However, since thermoplastics used in the manufacture of the temporal scaffolds present a melting point above the physiological temperature of living cells, which would endanger cell viability, the most feasible approach consists in the fabrication of scaffolds (3D printing) that can be cell seeded with a bioink a posteriori [26].

In this work, the influence of parameters of “hidden” importance in the design of PCL scaffolds, such as, number and orientation of layers, cooling down rate or positioning of the starting point in the printer, in the porosity and mechanical performance of the scaffolds, are studied. These parameters have been named “hidden” because they are not normally provided and, as it can be observed from the results, and discussed above, they may dramatically affect the performance and the reproducibility of thermoplastic scaffolds manufactured through 3D printing for bone tissue engineering. The ability to incorporate bioinks *a posteriori* has also been checked.

## 2. Materials and Methods

### 2.1. Materials

The PCL [(C_6_H_10_O_2_)_n_] used in this project was directly provided as a 1.75 ± 0.005 mm filament for standard 3D printers (3D4makers.com, Haarlem, Netherlands, *M*_w_: 84,500 ± 1000 Da, *ρ* = 1.145 g/cm^3^, 100% pure, yield stress for the raw material, 17.2 MPa, Young modulus, E = 470 MPa). 

The hydrogel employed for the filling of the scaffolds, for example, was an oral grade sodium hyaluronate (BioIberica, Barcelona, Spain) based gel [27].

### 2.2. Characterization of the Polymer

Thermal gravimetric analysis (TGA) was performed in a TA-Q500 (TA Instruments, Hüllhorst, Germany). A temperature range of 54–600 °C, with a heating rate of 10°/min and N_2_ atmosphere was used. Differential scanning calorimetry (DSC) was performed in a DSC 7 (Perkin Elmer, Waltham, MA, USA. A temperature range of −65–250 °C and a heating rate of 10°/min and N_2_ atmosphere was configured. The powder required for these characterizations was obtained by pulverizing filament pieces with a blade grinder in liquid nitrogen.

### 2.3. Scaffold Design and Fabrication

**Figure 1:**
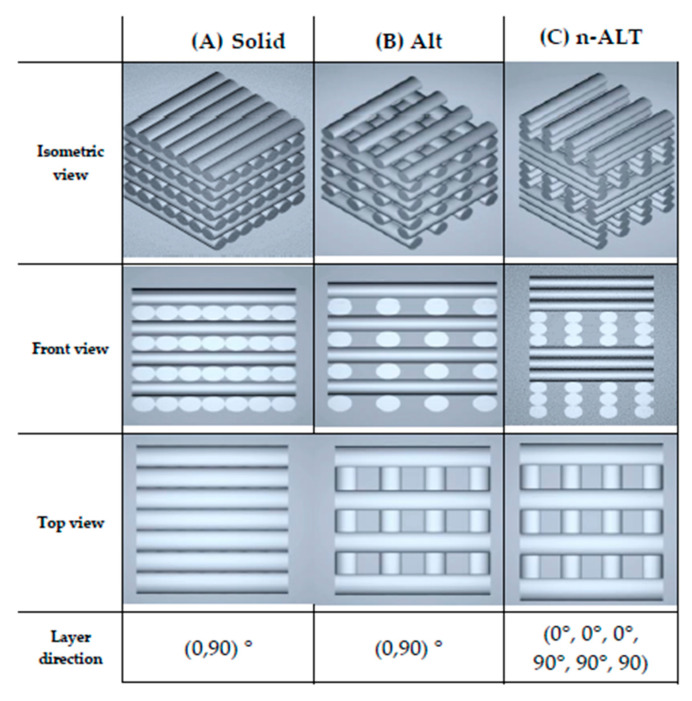
STL (Standard Tessellation Language) images of (**A**) solid (100% infill), and 30% (**B**) alternate (ALT) and (**C**) non-alternate (n-ALT) scaffolds.

Three different configurations were used for the design of the internal structure of the scaffolds, as shown in Figure 1; they are: a solid one with alternate layers (0°, 90°), a porous one with 30% infill and alternate layers (0°, 90°) (from now on, ALT) and finally another configuration where 3 layers are piled before changing the orientation (0°, 0°, 0°, 90°, 90°, 90°) also with 30% infill (n-ALT). All layer patterns were rectilinear and a layer height of 0.25 mm was used, unless stated otherwise. 

For the fabrication of the scaffolds, a Hephestos 2 (Prusa i3, BQ, Madrid, Spain) with a heated platform and a double drive gear extruder and a 400 μm nozzle was used. The nozzle temperature was set to 172 °C and the build plate to 40 °C. External geometries were designed for each analysis following the ISO normative indicated on each section. When noted (FAN), an external fan was employed to decrease the cooldown time (forced cool down specimens). The cooling down rate with and without the fan was experimentally measured, recording the temperatures obtained in the nozzle every 5 s after switching off the heating current. Without the fan, the cooling down rate described a non-linear curve that could be approximated to a second order polynomial (R^2^ = 0.999): 0.0223x^2^ − 3.5x + 182. Meanwhile, following the same behaviour but with a steeper initial rate, the cooling down rate of the system with the use of the external fan was modelled as (R^2^ = 0.993): 0.0223x^2^ − 3.5x + 182.

**Figure 2:**
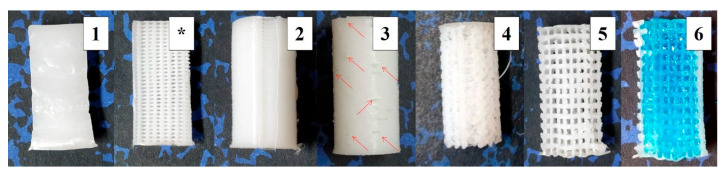
Pictures of the six different specimens: (**1**) Section of solid (100%) infill; (*) Section of the 30% alternated scaffolds [2,3]; (**2**) Aligned seam from fixed starting point at 30% ALT with shell; (**3**) Random starting point appearance at 30% ALT with shell; nonaligned defects pointed out with arrows; (**4**) Scaffold resulting from a slow cooling down at 30% n-ALT; (**5**) Forced cooling down (external fan) of 30% n-ALT; (**6**) 30% n-ALT filled with the hydrogel and blue colorant for a better contrast (from now 30% n-ALT+hyd).

Six different pattern probes were obtained from the three original designs (Figure 2): in the alternate configuration, the effect of the starting point position (aligned or random) of the layers was studied; in the case of the non-alternate configuration, scaffolds were printed first at room temperature (RT) and then with an external fan focused on the printed surface to study the effect of the cooling down speed. Finally, as the non-alternate scaffolds can also be printed in combination with cell-laden hydrogels to create composite materials in bioprinting, a final configuration was created filling the pores a posteriori with a hydrogel. Table 1 summarizes the parameters of the six patterns.

### 2.4. Scaffolds Characterization

#### 2.4.1. Microscopy: Optical and SEM

Images of the manufactured scaffolds were obtained with an optical microscope (Y-FL eclipse 400, Nikon, Tokyo, Japan). Pore morphology was determined by imaging cut samples using a XL30, scanning electron microscope (Phillips, Eindhoven, Netherlands), operating at 25 kV. Specimens were coated with Au-Pd using a Polaron SC7640 (Quorum Tech. LTD, Kent, UK) sputter coater. Fiji software (ImageJ v1.52h, U.S. National Institutes of Health, Bethesda, MD, USA) was used for image analysis [28]. 

#### 2.4.2. Mechanical Test

For the compression analysis, a QTest 1/L (Elite- MTS Systems Corporation Cary, North CA, USA) universal test machine was used. The load cell was configured for 1.000 N, with a maximum deformation of 20% at a speed of 5 mm/min. In this case, specimens were printed following the EN ISO 640 standard, as cylinders of 20 mm in height and with a diameter of 10 mm. As explained before in Section 2.3, six different specimens were tested (and for each one, the number of samples were six, n = 6). For each one, the compressive modulus of the scaffold, as well as the maximum load applied before breaking, were calculated.

#### 2.4.3. Dynamic Mechanical Analysis (DMA)

The viscoelastic properties and the complex modulus of the PCL filament were evaluated by dynamic mechanical thermal analysis (DMA) as a function of time/frequency and temperature using a DMA 861e (Mettler Toledo, Schwerzenbach, Switzerland). The dynamic heating scans were performed from −90 to 40 °C at 2 °C/min and 1, 3, 10 and 30 Hz. The static load strain and the dynamic load strain used in these experiments were 3 N (150%) and 2 N, respectively. For these analyses, the ISO 6721 protocol was followed for the printing of specimens: rectangles of 10 × 5 mm^2^ with a thickness = 50 μm and one perimeter of shell). 

#### 2.4.4. Statistical Analysis

GraphPad Prism 6 software (Graphpad Software, San Diego, CA, USA) was used for the statistical analysis. One-way analysis of variance (ANOVA) was performed with multiple comparisons to check for significant differences (*p* < 0.05).

## 3. Results

### 3.1. Polymer Characterization

The working temperature range for processing the material was studied through thermal analysis, TGA and DSC. T_g_, T_f_ and degradation temperatures can be found in Table 2. The TGA assay carried out within a 50–600 °C range showed a single thermal decomposition step starting at 392 °C and ending at 435 °C. Subsequent DSC analysis produced a T_g_ at −64 °C and a melting point at 51 °C that agree with literature values and should determine the processing temperature [29]. On the one hand, the PCL filament used has a melting point of 51 °C, and degrades above 392 °C; on the other hand, the work range of the printer can be set in the range (room temperature −230 °C), so the working temperatures that avoid compromising the integrity of both the polymer and the printer are (51–230 °C). Readers are referred to the discussion section for further explanations.

DMA analysis can be found in Supplementary Figure S1. PCL shows a prevalent elastic behaviour over 5 °C and up to 37 °C. Storage modulus decreases with the raise of temperatures. At physiological temperature, once the scaffold has been manufactured, the material holds an elastic modulus of 112.7 MPa (equal to the complex modulus) and a storage (viscous) modulus of 2.90 MPa.

### 3.2. Scaffolds Characterization

#### 3.2.1. Pore Size and Distribution 

**Figure 3:**
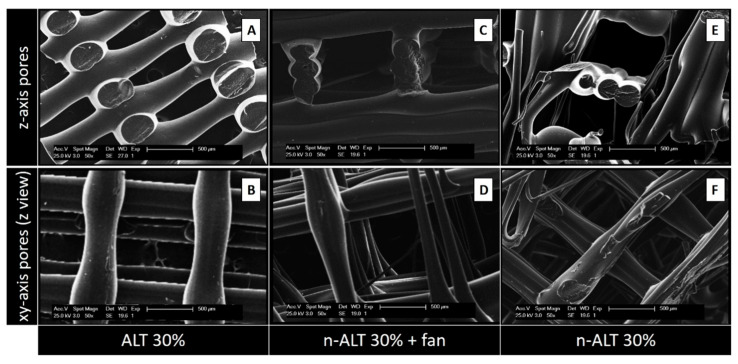
SEM images from the alternated (**A**,**B**) and non-alternated (**C**–**F**) scaffolds, obtained from lateral sections of cut samples (**A**,**C**,**E**) or from the top (**B**,**D**,**F**). E and F represent more chaotic structures due to the slow rate of cooling down, as no external fan was used. Scale bars: 500 µm.

SEM images from the alternated (A,B) and not alternated (C–F) scaffolds, obtained from lateral sections of cut samples (A,C,E) or from the top (B,D,F) can be found in Figure 3. Image A, that represents the most commonly used z-stack, shows a high anisotropic relation between the pore size in the z-axis when compared with the xy-pore (image B). While C shows a regular and ordered structure, due to a faster cooling down, image E reveals a more chaotic pattern, with some closed pores. The same effect can be observed from the top; in F, the polymer flowed a little bit before setting, creating flatter and thinner strands, in contrast with more cylindrical ones observed at D.

**Figure 4:**
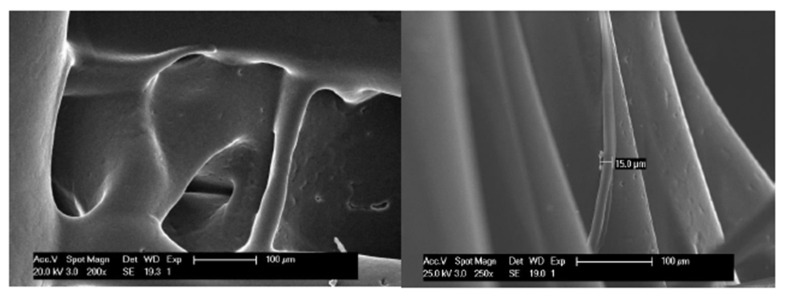
Micrograph of macro and micro pores and microfibres created randomly for a slow cooling down (not forced air flow) of the PCL (30% n-ALT). Scale bars: 100 µm.

In a more detailed view of the scaffolds with a slow rate of cooling down (30% n-ALT), we can observe the presence of random microfibers and pores with different sizes and morphologies (Figure 4). 

**Figure 5:**
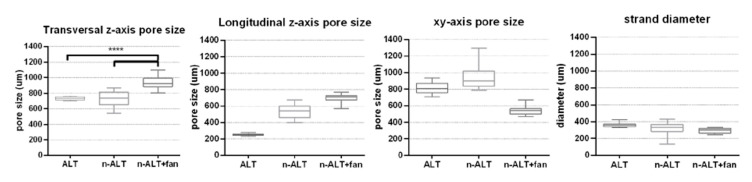
Box and whiskers plot for pore sizes measured with ImageJ at z- axis (transversal = horizontal, longitudinal = vertical) and at xy-axis (in any direction between parallel walls), along with the strand diameter. (n = 3 samples with at least 4 measurements per sample, mean ± SD, **** *p* < 0.001).

From the six initial configurations, pores and strand sizes were measured with ImageJ from the obtained SEM images of the three different scaffolds with 30% infill (samples 1, 3 and 4) and the results can be found in Table 3 and Figure 5. No significant differences can be observed between the strand size from ALT to n-ALT, whereas in the case of n-ALT+fan, it is slightly thinner than ALT. When the cooling down is quick, the resulting filament sets faster, generating a more cylindrical strand that results in a regular, open-pore structure. On the one hand, this is a positive effect as it avoids pore collapsing, generating a more open scaffold with higher shape fidelity. However, it also leads to a smaller surface contact between layers and the fast cooling down of the polymer can also generate a more brittle construction. Scaffolds with alternate strands display lower squared z-pore morphologies, whereas n-ALT specimens possess a higher heterogeneity between specimens.

#### 3.2.2. Compression Test

**Figure 6:**
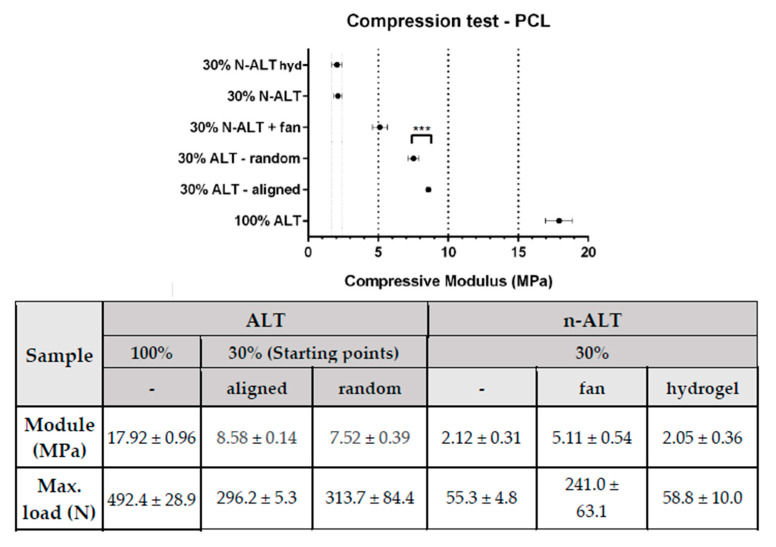
Summary of the compression test results (± standard deviation) with a comparative graph at the top. (n = 10, mean ± SD, *** *p* < 0.0002). All, but the first two (w and w/o hydrogel), represent significative difference with *p* < 0.0001.

Compression test results have been summarized and represented in Figure 6. As expected, solid samples (100% ALT) showed the highest compressive modulus (~18 MPa), with a reduction of more than a half when the infill was reduced to 30%. 

Among the samples with a 30% of infill, scaffolds with alternate strands (ALT) held up higher stresses than those where the direction of the strand was changed after three layers (n-ALT). Among the non-alternate configurations (n-ALT), a higher compressive modulus was found when the cooling down temperature was forced by the use of the external fan (n-ALT +fan). Non-alternate structures (n-ALT) have bigger z-axis pores, as they present more transversal strands. Because of this, the n-ALT configuration leads to an easier layer collapse, weakening the scaffold, as can be shown in the reduction in the compressive modulus.

When no fan was used, a less ordered structure was obtained than with forced cool down. When samples with aligned (30% ALT-aligned) and random (30% ALT-random) starting points are compared (Figure 2, samples 2 and 3) a slightly, but significant (*p* < 0.0002) difference in the compressive module is found. Finally, when a hydrogel was used to fill the gaps inside the scaffolds (n-ALT +hyd), no significant change (with n-ALT) was observed. 

## 4. Discussion

Since the PCL filament used has a melting point of 51 °C, but only degrades above 392 °C, the working range of the printer should be set in a range that does not compromise the integrity of the polymer or the printer (52–230 °C). The recommended temperatures to print provided by the manufacturer are in a shorter range: 115–145 °C. However, due to the technical restrictions of our 3D Printer, the minimum printing temperature was 172 °C. It provided us with an appropriate polymer fluency during the printing, but the need of an extra fan to obtain a proper cooling down rate. Although it is a high value, we work inside the theoretical safe range of the polymer, decreasing the chance of PCL oxidation, as it has been reported in the literature that the oxidation onset temperature for pure PCL is 240.5 °C [30]. 

Adhesion of the specimen to the heated platform, and then heated platform temperature and cool down conditions, will determine the appropriate printing temperature in each case. Our heated platform was set at 40 °C, within the range of 30–45 °C provided by the manufacturer. The reason for this selection was that lower temperatures led to a poor adhesion to the heated platform and the impossibility of building up in the z-axis, where higher temperatures could lead to cell damage in the eventual intended application. 

The measured storage modulus is indicative of the elastic energy stored in the material, and it is affected by changes in the morphology of the material, induced, for example, by the printing process. The material holds a storage modulus of 123 MPa at physiological temperature and decreases with an increase in temperature and lower values than those provided by the manufacturer, which is indicative of the influence of the processing and described in the literature as related to the effects of the elongation in the crystallinity of the polymer [16]. The evaluation of crystallinity is outside of the scope of this work since this analysis would require synchrotron radiation for an effective analysis. 

More interesting is the value obtained for the viscous modulus (2.90 MPa). It is generally acknowledged that mechanostructural stimuli from the surrounding microenvironments of cells crucially influence cellular functions [31]. Later research focused on the viscous modulus, since the elastic modulus could not fully account for the different cell responses observed to different substrates. It has been found that cells are significantly more sensitive to the fluidic motion of a substrate than to the elastic modulus, especially for biodegradable scaffolds [32], and a minimum in the complex modulus should be the most favourable condition for tissue culturing in these scaffolds as obtained for our printed PCL.

Then, in addition of the intrinsic properties of the thermoplastic selected for the manufacturing of the scaffold, cool down conditions and design parameters determine the properties of the printed device. An important factor in the final characteristics of the obtained scaffolds, frequently ignored in the literature, is the cooling down conditions. When the pictures and micrographs of the scaffolds, shown in Figure 3, are compared; E, F for the slow cooled down to room temperature scaffolds, and C, D for the forced cool down scaffolds, it can be clearly appreciated how scaffolds C, D present a regular and homogeneous pattern, while scaffolds E, F show a rather chaotic structure including some collapsed pores and a major heterogeneity for fibre diameters. It can be deduced that when the cooling down is quick, the resulting filament sets faster, generating a more cylindrical strand that yields a regular, open-pore structure. On the one hand, this could be something desirable as it avoids pore collapsing, generating more open porosity and higher shape fidelity. On the other hand, it may lead to a smaller surface contact between layers that should yield a more brittle construction. Moreover, it can be observed that when the pore sizes are significantly bigger (Figure 3C,D), this increase in the distance and the reduction in transversal layers leads to a smaller modulus and ultimate strength. However, it is important to notice that although this heterogeneity involved lower mechanical properties, it may be favourable for the biological performance, since it provides a better environment for cell attachment and proliferation [33]. 

The surface and the general texture of the constructs were also altered due to the fast/slow cooling down rate, as it can be appreciated in the SEM images displayed in Figure 4. Slow cooling creates different roughness and triggers the apparition of small random internal strands. Although at first view those lines could be considered as defects, it has been described that greater roughness yields better cell attachment and proliferation [34]. This behaviour can be attributed to the rubbery character of PCL at room temperature, since its T_g_ is −60 °C. Therefore, this phenomenon can be different from other thermoplastic polymers also used for bone engineering, such as poly(glycolic) acid (PGA), T_g_ 35–40 °C or poly(lactic) acid (PLA) T_g_ (55,2 °C) [35] and different copolymers [36]. This suggests that the T_g_ may have a role to play in the final features of the scaffolds and it must be considered in the decision whether to force down the cooling rate in the design of the manufacturing parameters.

It is usually considered that FDM printing produces well defined porous structures [37], but is not suitable for cell attachment and proliferation. Electrospinning [38], low-temperature fused deposition modelling techniques [39] or melt electrospinning [40] are then incorporated as complementary techniques to provide fibres that can be used as substrate and guides for cell proliferation. However, as shown in this work, a chaotic structure of fibres that can be used as substrates can also be obtained by simply controlling the cooling rate and printing temperature without the need of implementing further techniques, showing that with a deep knowledge and careful control of printing parameters it is possible to build up scaffolds appropriate for bone engineering.

The manufacturing of porous scaffolds for tissue ingrowth also requires that porosity is interconnected for the ingrowth of the tissue and vascularization that avoids hypoxia and failure of the constructs [41]. Thus, it is important in the design of porosity along all directions. In this work, infill was set at 30% for being an appropriate porosity percentage for fulfilling these characteristics, and to obtain a similar xy and z-pore size. Moreover, this porosity percentage is described as close to the optimized to obtain a homogeneous cell distribution when seeded [42] and no further experiments were needed. From there, two different approaches can be taken, alternate and non-alternate layers, as can be visualized in Figure 1. Alternate scaffolds offer larger pores in *x* and *y* directions, increase the space for media and nutrients flow, cell spreading, tissue ingrowth and vascularization, although they have yielded lower mechanical properties and a small pore size along the z-axis can also lead to the collapse of the pores, compromising the flow and tissue ingrowth. 

Tissue growing studies in three dimensions present several limitations. Flow paths through the porosity of the scaffold should be managed to ensure that the concentration of dissolved nutrients and cellular by-products is maintained within limits suitable for viable cell culture [43]. The flow of culture fluid exposes cells to hydrodynamic shear, which has been shown to be a significant factor in regulating cellular behaviour [44]. The micro-architecture of a porous structure has a significant effect on the local shear stress distribution. Even with a regular average fluid shear stress through the structure, local maximum, and minimum variations will be present depending on the geometry of the pores. Minimization of the variation between local shear stresses is an important consideration for scaffold design, as large local variation may cause undesired cellular behaviour even when the average shear stress is at an optimal level [23].

However, accurately modelling fluid flow through highly porous structures tends to be extremely computationally intense due the large amount of surface to fluid interactions and the challenge of refining the calculations to a resolution that sufficiently represents the real geometry [45]. On the other hand, a significant factor limiting further progress in the development of large and complexly shaped tissue scaffolds is the absence of readily available equipment and methods to measure fluid field properties and scaffold–tissue geometry at a cellular resolution [23]. The development of techniques with improved resolution, depth of analysis and greater range of discernible tissue is a vital step for advancing our ability to engineer large and anatomically accurate tissue scaffolds. All these efforts are completely outside of the scope of this manuscript. 

The printing starting point, which in principle should not affect pore or strut size or the properties of the manufactured scaffold, is a parameter that must be set when designing scaffolds. Our measurements indicate that it may have an effect on the compression modulus of the scaffolds being greater when an aligned design is defined. This may explain the better mass distribution in aligned designs than in random set designs. Although at first thought the samples with aligned starting points seem to be the weakest, as they present a preferential path for defect propagation, they hold a higher modulus than those specimens where the starting point was random. One possible explanation for this would be that the random pattern requires longer movements between layers, which can create oozing (material leaking) and lead to a thinner starting strand or even zones with no polymer deposition. This issue could be optimized by decreasing retraction, but this will also avoid the formation of microfibres. Of course, this effect is more visible when printing with an external shell that will also act as the main supporting load zone.

Scaffolds filling with the hydrogel have been effective, as shown in supplementary images 2 and 3. The hydrogel fills in all the spaces without introducing defects, and, consequently no significant changes were observed in the compression properties in comparison with the unfilled analogous scaffold (this is expected, as PCL is stiffer than the hydrogel and supports all the load). This is indicative that the design and printing parameters selected are suitable for both the most common current approach of adding a cell seeded hydrogel *a posteriori* but also offer potential for a simultaneous printing of scaffold and bioinks for advance bioprinting [46,47].

## 5. Conclusions

There are several printing parameters, not usually reported, that may affect the performance of 3D-printed scaffolds. Other than the porous design, these parameters include cooling down rate, the use or not of alternate layers, the alignment of the printing starting point and the infill with a complementary gel.

The cooling down rate is an important parameter for the final characteristics of the scaffold. Forcing cooling down leads to higher internal and external shape fidelity in scaffolds made with polymers whose T_g_ is under room temperature, as in PCL. The cooling down rate, or even printing speed, can be modified to obtain more chaotic micropatterns including nanofibers without the need of using further technologies. 

The use of alternated layers involves the generation of more anisotropic structures, as the z-axis pore is limited to the layer height, while the x,y-pore size can be tailored to specific needs. The defects created because of the alignment, or the lack of it, in the new layer starting point, significantly affects the mechanical behaviour of the scaffolds.

## Figures and Tables

**Table 1:** Summary of probes configurations (specimens) and their intended features.

n	Infill	Layers	Sample Abbrv.	Other	Notes
1	100%	ALT	100% ALT	solid	Original cylinder for comparison, no pores.
2	30%	ALT	30% ALT-aligned	aligned starting point	Porous scaffold, with anisotropy in the Z-axis Aligned start point (seam)
3			30% ALT-random	random starting point	Porous scaffold, with anisotropy in the Z-axis Random start point
4	30%	n-ALT	30% n-ALT	RT cool down	Porous scaffold, with more isotropy in the Z-axis Slow cooling down (room temperature)
5			30% n-ALT+fan	external fan	Porous scaffold, with isotropy in the Z-axis Forced cooling down (external fan)
6			30% n-ALT+hyd	filled - hydrogel	Porous PCL scaffold, filled with crosslinked hydrogel

**Table 2:** Thermal characterization of PCL. T_g_ is the glass transition temperature, t_f_ the melting point, and T_deg_ the degradation temperature.

	T_g_	T_f_	T_deg_
**Ref. T [7]**	−62 °C	55–60 °C	-
**TGA**	-	-	392–435 °C
**DSC**	−64 °C	51 °C	-
**DMA (10 Hz)**	−61 °C	53 °C	-

**Table 3:** Pore and strand size distribution (values represented by its means ± SD, standard deviation) for 30% ALT, n-ALT (room temperature cooling down, RT) and n-ALT forced cool down (+fan). In addition, the coefficient of variation of the results (x%) was included for each measurement.

Sample	z-axis			xy-axis Pore Size (μm)	Strand Diameter (μm)	Homogeneous Distribution? (Pore and Strand)
	Pore Size (μm)		Trans. vs. Long. Ratio			
	Transversal	Longitudinal				
**30% ALT**	733 ± 22 (3%)	254 ± 13 (5%)	2.90 ± 0.24 (8%)	816 ± 61 (8%)	361 ± 26 (7%)	✓—huge anisotropy
**30% n-ALT+ fan**	938 ± 80 (9%)	689 ± 60 (9%)	1.37 ± 0.22 (16%)	542 ± 48 (9%)	290 ± 30 (10%)	✓—more isotropic pores in z-axis
**30% n-ALT (RT)**	728 ± 90 (12%)	532 ± 87 (16%)	1.43 ± 0.40 (27%)	930 ± 123 (13%)	314 ± 87 (28%)	✗—higher variability

## Supplementary Materials

The following are available online at https://www.mdpi.com/2073-4360/12/7/1546/s1, Figure S1: DMA curves for PCL printed samples at 10 Hz, to obtain the Tg of the polymer, as well as its viscoelastic properties as a function of the increasing temperature. On top, all the studied range is shown. At the bottom, a detailed view of the linear viscoelastic region is shown. Stamped values of storage (elastic), loss (viscous) and complex modulus were added at the beginning and at the end of the linear viscoelastic region.; Figure S2: Photographs and SEM images of the internal (section) of a composite formed by the scaffold and the hydrogel. Magnification included as head in each column. Cracks observed can be attributed to the contraction from the drying process. Similar pictures from the top, and from the individual materials of the composite can be found in Supp. Figure 3; Figure S3: SEM images with different magnification zooms of (from top to bottom): empty scaffold, hydrogel, top view of the composite formed by the scaffold and the hydrogel, and internal view of this composite obtained from a section. Magnification included as head in each column.
