# Supplementary material for: Design of Thermoplastic 3D-Printed Scaffolds for Bone Tissue Engineering: Influence of Parameters of “Hidden” Importance in the Physical Properties of Scaffolds
Source: Polymers (Basel). 2020 Jul 13;12(7):1546. doi: 10.3390/polym12071546 (PMC7408024; doi:10.3390/polym12071546)
Supplement: Supplementary file 1 [file polymers-12-01546-s001.pdf]

Design of Thermoplastic 3D-Printed Scaffolds for Bone Tissue Engineering: Influence of Parameters of “Hidden” Importance in the Physical Properties of Scaffolds

Nieves Cubo-Mateo [1,2] and Luis M. Rodríguez-Lorenzo [2,*]

[1] Sensors and Ultrasonic Systems Department, Institute for Physical and Information Technologies, ITEFI-CSIC, Madrid, Spain; nieves.cubo@csic.es
[2] Department of Polymeric Nanomaterials and Biomaterials, ICTP-CSIC, Madrid, Spain
* Correspondence: luis.rodriguez-lorenzo@ictp.csic.es

Received: 4 June 2020; Accepted: 9 July 2020; Published: date

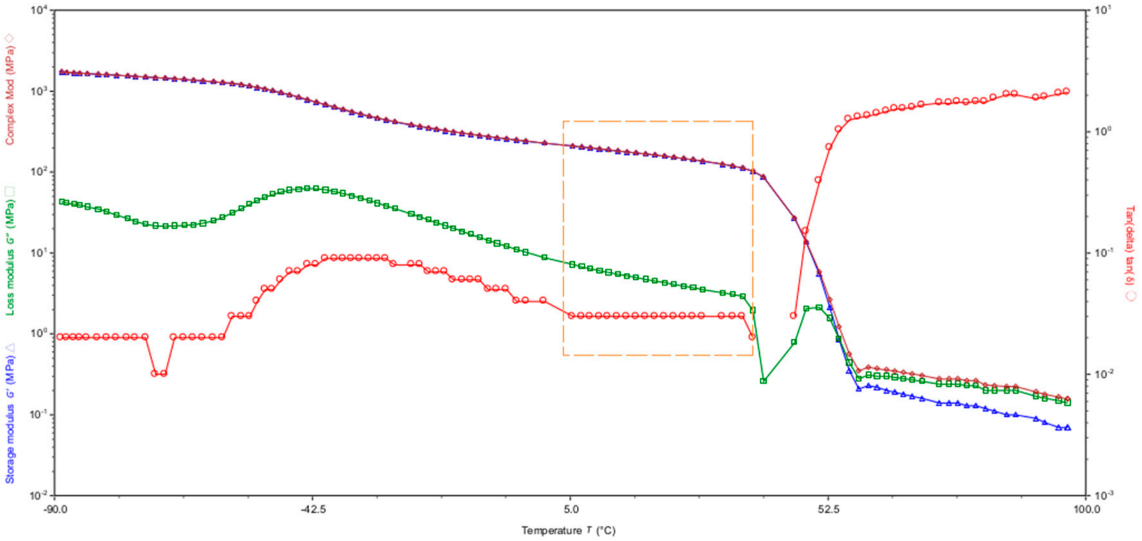

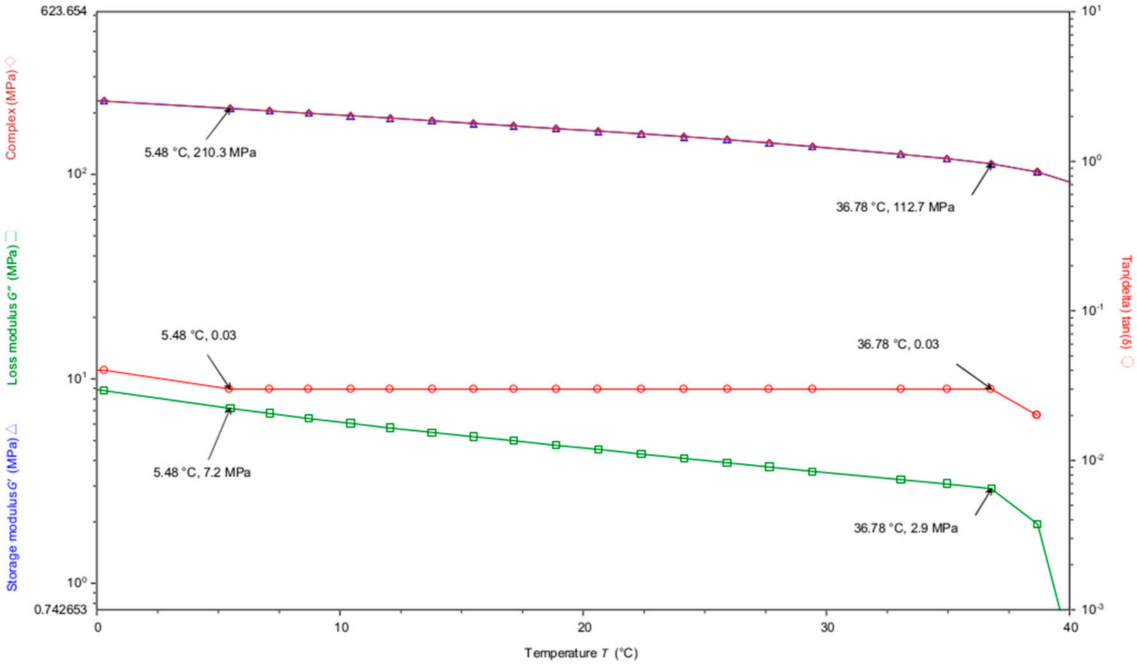

Figure 1. DMA curves for PCL printed samples at 10 Hz, to obtain the Tg of the polymer, as well as its viscoelastic properties as a function of the increasing temperature. On top, all the studied range is shown. At the bottom, a detailed view of the linear viscoelastic region is shown. Stamped values of storage (elastic), loss (viscous) and complex modulus were added at the beginning and at the end of the linear viscoelastic region.

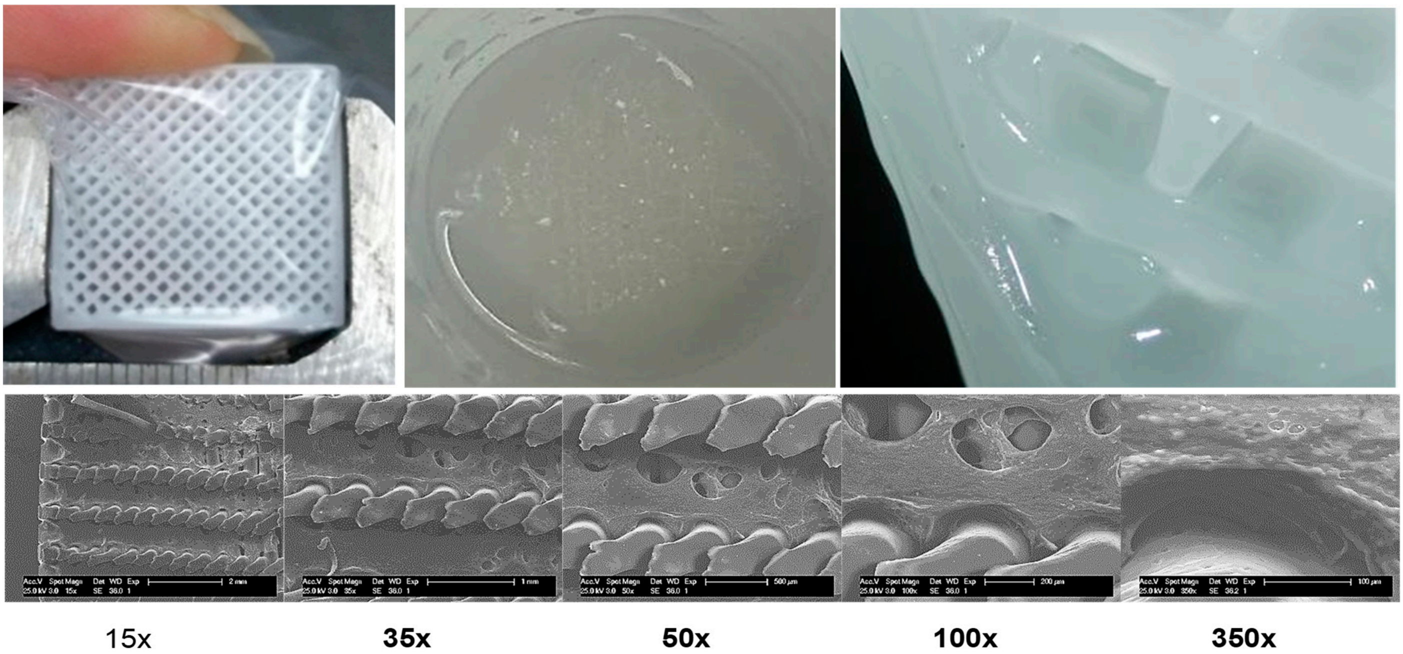

Figure 2. Photographs and SEM images of the internal (section) of a composite formed by the scaffold and the hydrogel. Magnification included as head in each column. Similar pictures from the top, and from the individual materials of the composite can be found in Figure S3.

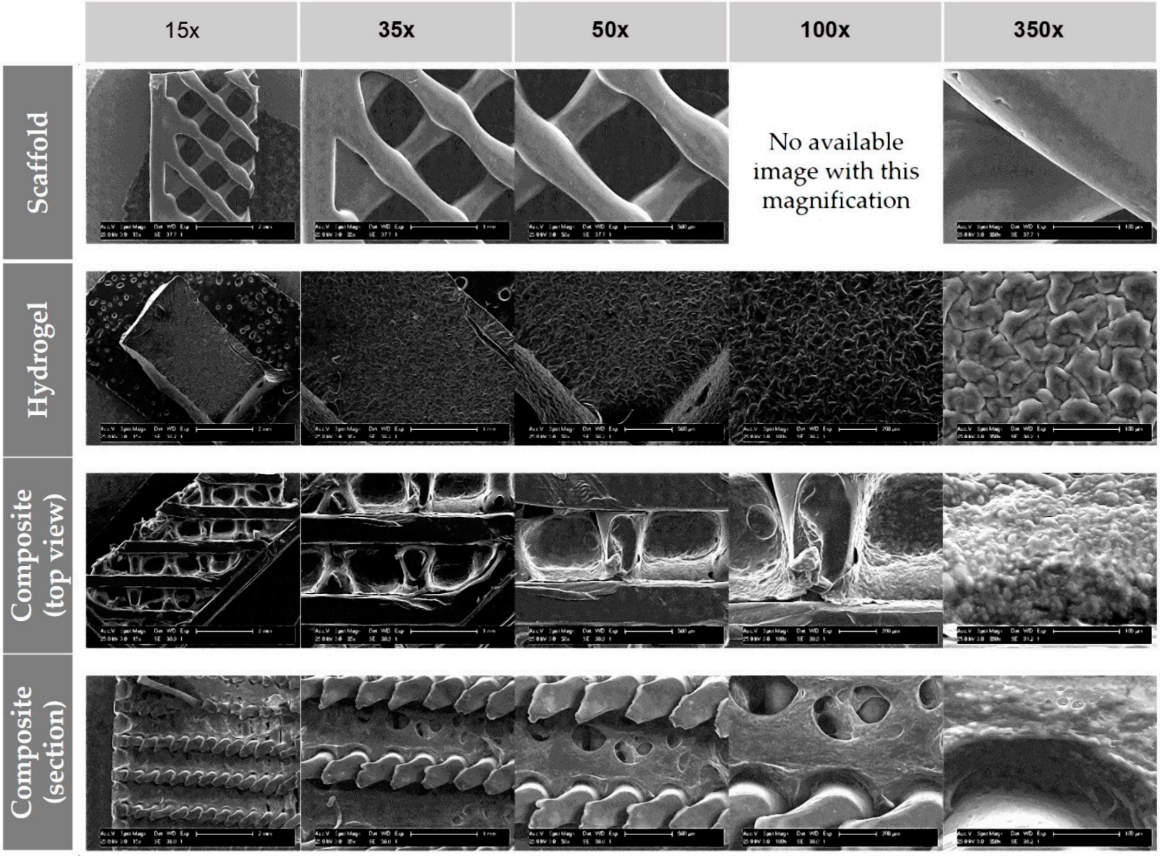

Figure 3. SEM images with different magnification zooms of (from top to bottom): empty scaffold, hydrogel, top view of the composite formed by the scaffold and the hydrogel, and internal view of this composite obtained from a section . Magnification included as head in each column.
